# Regulation of Disease-Resistance Genes against CWMV Infection by NbHAG1-Mediated H3K36ac

**DOI:** 10.3390/ijms25052800

**Published:** 2024-02-28

**Authors:** Aizhu Tu, Mila Wu, Yaoyao Jiang, Lidan Guo, Yunfei Guo, Jinnan Wang, Gecheng Xu, Jingjing Shi, Jianping Chen, Jian Yang, Kaili Zhong

**Affiliations:** State Key Laboratory for Managing Biotic and Chemical Threats to the Quality and Safety of Agro-Products, Key Laboratory of Biotechnology in Plant Protection of Ministry of Agriculture and Rural Affairs and Zhejiang Province, Institute of Plant Virology, Ningbo University, Ningbo 315211, China; tuuu414@163.com (A.T.); milaaa2018@163.com (M.W.); yyaojiang@163.com (Y.J.); dan750423443@163.com (L.G.); gyf835492187@163.com (Y.G.); 15258141571@163.com (J.W.); xxxgccc@163.com (G.X.); 18958360656@163.com (J.S.); jianpingchen@nbu.edu.cn (J.C.)

**Keywords:** Chinese wheat mosaic virus, histone acetylation, NbHAG1, transcriptome, disease-resistance pathways, *Nicotiana benthamiana*

## Abstract

Post-translational modification of proteins plays a critical role in plant–pathogen interactions. Here, we demonstrate in *Nicotiana benthamiana* that knockout of NbHAG1 promotes Chinese wheat mosaic virus (CWMV) infection, whereas NbHAG1 overexpression inhibits infection. Transcriptome sequencing indicated that a series of disease resistance-related genes were up-regulated after overexpression of NbHAG1. In addition, cleavage under targets and tagmentation (Cut&Tag)-qPCR results demonstrated that NbHAG1 may activate the transcription of its downstream disease-resistance genes by facilitating the acetylation level of H3K36ac. Therefore, we suggest that NbHAG1 is an important positive regulator of resistance to CWMV infestation.

## 1. Introduction

Plant cells perform their complex physiological functions mainly through proteins. Protein post-translational modifications (PTMs) fine regulate protein structure and therefore their function, localization, activity and protein–protein interactions [1,2]. Protein acetylation is a ubiquitous PTM in both eukaryotes and prokaryotes and is involved in a variety of key cellular processes, including DNA replication, gene expression, protein translation, enzymatic activity, protein stability, and cell signaling [3,4]. Acetylation is reversible with histone acetyltransferases (HATs) and histone deacetylases (HDACs) acting synergistically to maintain endogenous histone acetylation homoeostasis [5,6]. GENERAL CONTROL NON-REPRESSIBLE 5(GCN5, also known as histone acetyltransferase of the GNAT family 1, HAG1) is a well-studied histone acetyltransferase that plays an essential role in development in mice [7]. It is closely linked to gene transcriptional regulation since deleting GCN5 results in the up- or down-regulation of a large number of genes in yeast [8]. GCN5 is required for normal developmental processes in Drosophila, which carries a null allele that exhibits defects in metamorphosis and oogenesis [9]. GCN5 forms a conserved multi-subunit SAGA (Spt-Ada-Gcn5 acetyltransferase) complex in yeast, metazoans, and plants [10,11]. GCN5 is evolutionarily conserved and an ortholog HAG1 was found in Arabidopsis [12]. AtGCN5/HAG1 appears to be an important HAT required for gene expression changes involved in numerous plant development pathways and responses to environmental conditions in Arabidopsis [13,14]. AtGCN5-dependent histone acetylation products H3K9ac, H3K14ac and H3K27ac are required for the expression of a large number of genes, suggesting that GCN5 is involved in both long-term epigenetic regulation of chromatin modifications and short-term control of transcriptional switches [15]. The Wuschel-associated homeobox gene WOX11 in rice recruits the ADA2-GCN5 histone acetyltransferase module to activate downstream target genes (such as QsPIN9, OsCESA9, OsGLU5) in crown root meristem, which are involved in energy metabolism, cell wall biosynthesis, and hormone responses [16]. In addition, GCN5 mediates plant adaptation to environmental and stress signals, including light, DNA damage, phosphate starvation, iron homeostasis, and salt, heat and cold stresses [17,18].

Chinese wheat mosaic virus (CWMV) is a soil-borne virus that is one of the major pathogens causing wheat mosaic disease and was first described in China [19]. CWMV is transmitted by *Polymyxa graminis*, which is a specialized parasite on the roots of graminaceous plants [20]. CWMV is classified in the genus *Furovirus* in the family *Virgaviridae* and has a genome consisting of two single-stranded positive-sense RNAs encapsidated in rigid rod-shaped particles [21]. In older leaves, CWMV infection appears as bright yellow chlorotic streaks and even purple chlorotic streaks. In severe infections, the plant becomes stunted, wilted, and eventually dies [22]. Full-length cDNA clones of CWMV previously constructed have proved infectious on wheat (*Triticum aestivum*) and the model plant *Nicotiana benthamiana*, by mechanical inoculation [23].

Histone acetyltransferases like GCN5, also known as HAG1, are regulators of gene expression and play diverse roles in cellular processes across eukaryotes. They mediate reversible acetylation of histones, impacting transcriptional activation and repression. While extensively studied in development and stress responses, their involvement in viral infection, particularly in wheat, remains poorly understood. This study investigates the functional role of NbHAG1 during CWMV infection. By analyzing changes in gene expression related to disease resistance pathways, it elucidates a regulatory mechanism wherein NbHAG1 modulates gene expression through H3K36 acetylation, shedding light on histone acetylation dynamics during viral infection.

## 2. Results

### 2.1. CWMV Infection Induces HAG1 Expression in N. benthamiana

**Figure 1:**
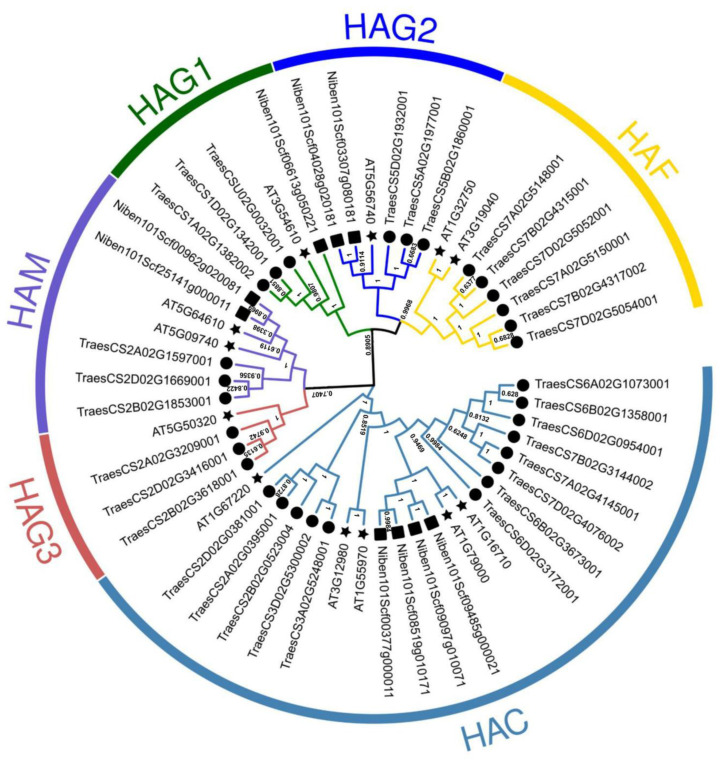
Phylogenetic analysis of the HAT proteins. Phylogenetic tree of HAT proteins from *A. thaliana*, *T. aestivum,* and *N. benthamiana* constructed by the neighbor-joining method in MEGA-X. The numbers at nodes represent bootstrap values after 1000 iterations. Each group is indicated by a different color. Stars represent *A. thaliana*, circles represent *T. aestivum*, and squares represent *N. benthamiana*.

**Figure 2:**
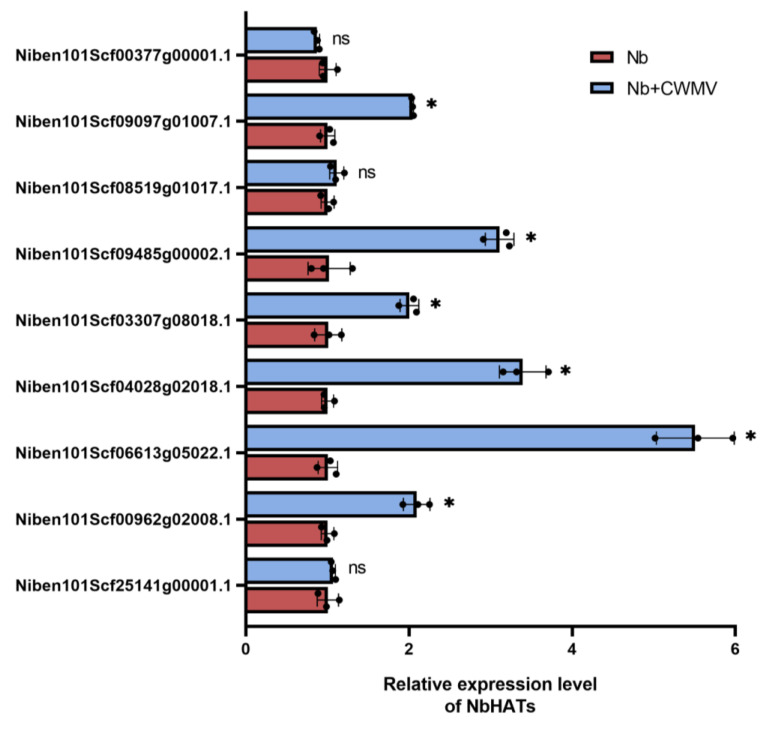
CWMV infection induces HAG1 expression in *N. benthamiana*. Relative expression levels of TaHATs from plants inoculated with CWMV measured by qRT-PCR at 14 dpi. Data presented are the mean ± SD of three biological samples per treatment. Each biological sample had three technical replicates. Significant differences between treatments were determined using Student’s *t* test (*, *p* < 0.05, ns, not significant).

Previous studies have shown that protein acetylation is enhanced by CWMV infection. In particular, the level of acetylation at multiple sites of H3 was significantly up-regulated in CWMV-infected plants [24]. Whereas the acetylation process is co-regulated by acetylases and deacetylases, the expression level of *TaHATs* continued to increase from 7 to 16 days post inoculation (dpi) along with an increase in CWMV accumulation [25]. Previously, we constructed infectious full-length cDNA clones of CWMV in the laboratory and successfully infected *T. aestivum* and *N. benthamiana* by mechanical inoculation [23], so we first investigated the role of HATs in CWMV infection of *N. benthamiana*. In order to identify the HAT family members in *N. benthamiana*, we used HAT protein sequences from the *A. thaliana* and *T. aestivum* genomes as a query, and identified a total of nine HAT protein sequences in the *N. benthamiana* genome database using BLAST (https://www.solgenomics.net/ftp/benthamiana/annotation/Niben101/, accessed on 13 August 2023) (Table S1). To analyze the phylogenetic relationships among HATs from different species, 12 *A. thaliana* (diploid), 31 *T. aestivum* (hexaploid), and nine *N. benthamiana* (tetraploid) HAT protein sequences were used to construct a neighbor-joining (NJ) tree. Unrooted trees that make no assumptions about ancestry illustrate only the relationships among the nodes [26]. Our analysis divided the HAT proteins from the three species into six branches for HAC, HAF, HAM, HAG1, HAG2, and HAG3 with 22, 8, 7, 5, 6, and 4 members, respectively (Figure 1). The NbHAT proteins were highly homologous to those from other species. They clustered into the same clades as AtHATs and TaHATs with high bootstrap support values. We then investigated the expression levels of the nine *HATs* in *N. benthamiana* after CWMV infection, using the empty vector mock-inoculated *N. benthamiana* as control. The expression levels of six *NbHATs* were significantly increased in the infected plants 14 days post-inoculation compared to the mock-inoculated controls (Figure 2). Niben101Scf06613g05022.1 had the most significant increase in expression, and we therefore used this for further study, naming it *NbHAG1*.

### 2.2. NbHAG1 Positively Regulates N. benthamiana Resistance to CWMV

**Figure 3:**
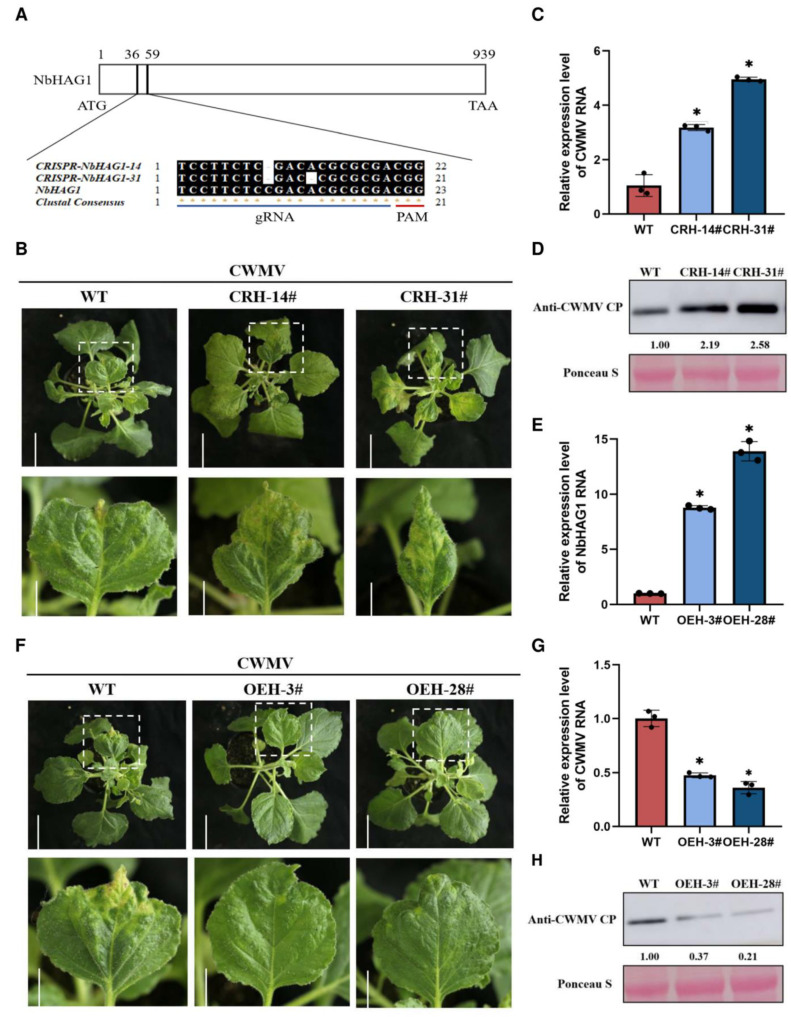
NbHAG1 positively regulates *N. benthamiana* resistance to CWMV. (**A**) Schematic representation of CRISPR/Cas9 to knockdown *NbHAG1*. The red line region indicates the PAM sequence (Protospacer Adjacent Motif) for the target DNA sequence that Cas9 will bind or cleave; the blue line region indicates the gRNA (guide RNA) for recognizing the target genomic sequence. (**B**) Phenotypes of *NbHAG1* knockout mutants and WT plants after CWMV infection. Symptoms were observed and photographed on day 21 after CWMV inoculation. Scale bars = 5 cm (upper panel) or 2 cm (lower panel). (**C**) The expression of CWMV RNA in *NbHAG1* knockout mutants and WT plants was examined by qRT-PCR. (**D**) Western blot detection of the protein content of CWMV CP in *NbHAG1* knockout mutants and WT plants. CWMV was inoculated on *NbHAG1* knockout mutant plants and the samples were taken at 14 dpi, and the content of CWMV CP was detected by Western blotting. The protein content in the different samples was then determined by Ponceau S. Viral proteins were quantified using ImageJ software. (**E**) The expression of *NbHAG1* in *NbHAG1* overexpression mutant plants was examined by qRT-PCR. (**F**) Phenotypes of *NbHAG1* overexpression mutants and WT plants after CWMV infection. Symptoms were observed and photographed on day 21 after CWMV inoculation. Scale bar = 5 cm (upper panel), scale bar = 2 cm (lower panel). (**G**) The expression of CWMV RNA in *NbHAG1* overexpression mutants and WT plants was examined by qRT-PCR. OEH means OENbHAG1. (**H**) Western blot detection of the protein content of CWMV CP in *NbHAG1* overexpression mutants and WT plants. CWMV was inoculated on *NbHAG1* overexpression mutant plants and the samples were taken after 14 dpi, and the protein content of CWMV CP was detected by Western blotting. The protein content in the different samples was then determined by Ponceau S. Viral proteins were quantified using ImageJ software (Version: 1.8.0). Data presented are the mean ± SD of three biological samples per treatment. Each biological sample had three technical replicates. Significant differences between treatments were determined using Student’s *t* test (*, *p* < 0.05).

To investigate the mode of action of NbHAG1 during CWMV infection, we used the CRISPR/Cas9-mediated genome-editing tool to knockout *NbHAG1* and obtained two stable lines (CRH-14# and CRH-31#) (Figure 3A). CWMV was then inoculated onto these plants and wild type (WT) controls by agroinfiltration. At 21 dpi, CRISPR NbHAG1 plants showed more serious CWMV symptoms than WT plants (Figure 3B). At 14 dpi, quantitative reverse transcription-PCR (qRT-PCR) and Western blot results showed that the accumulation of CWMV genomic RNAs and CP protein was significantly higher in CWMV-inoculated CRH-14# and CRH-31# than in WT plants (Figure 3C,D). We also obtained two stable *N. benthamiana* lines overexpressing HAG1 (OEH-3# and OEH-28#) at levels 8.78 and 13.91 times higher than that the wild type, respectively (Figure 3E). After inoculation with CWMV by agroinfiltration, OEH-3# and OEH-28# plants had weaker mosaic symptoms than WT plants at 21 dpi (Figure 3F). Moreover, qRT-PCR showed that the accumulation of CWMV genomic RNAs in the systemic leaves of the OENbHAG1 plants was significantly reduced in comparison with the WT (Figure 3G). Similar results were also obtained by Western blot analysis using a CWMV CP specific antibody (Figure 3H). All these results suggested that NbHAG1 negatively regulates *N. benthamiana* resistance against CWMV. There were no significant phenotypic effects of NbHAG1 knockout or overexpression (Figure S1). We speculated that NbHAG1 might inhibit CWMV infection by interacting with the CWMV viral proteins but no direct interaction between NbHAG1 and any of the CWMV proteins was found by yeast two-hybrid assay (Figure S2).

### 2.3. NbHAG1 Affects the Acetylation Level of H3K36

**Figure 4:**
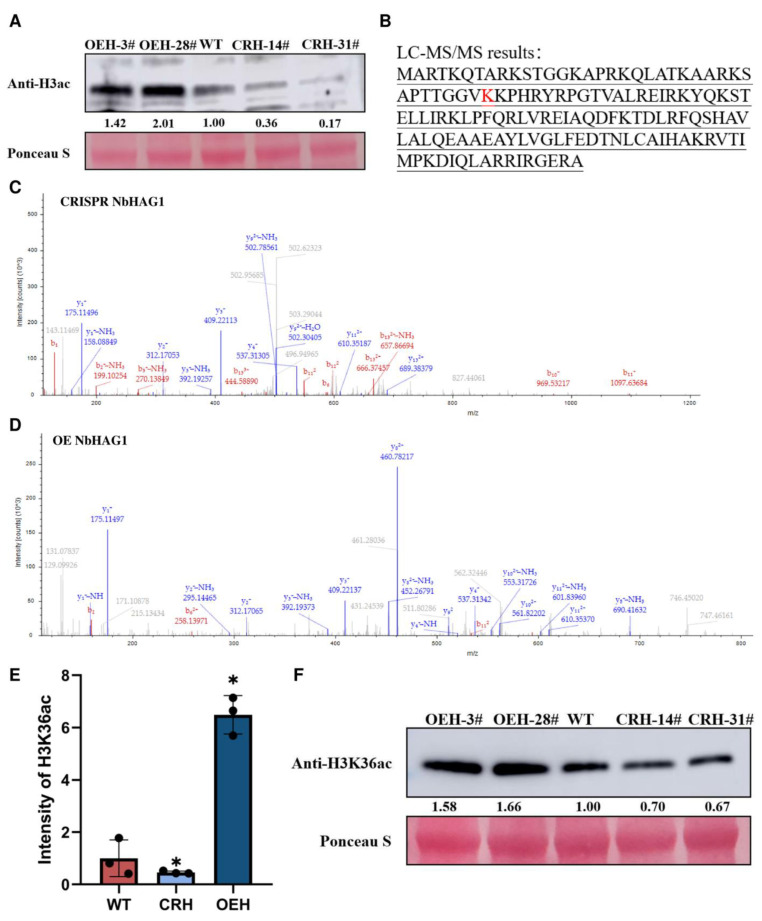
NbHAG1 mediates H3K36ac to regulate downstream gene expression. (**A**) Western blot detection of the protein content of H3ac in *NbHAG1* knockout mutants, WT and *NbHAG1* overexpression mutant plants. The protein content in the different samples was then determined by Ponceau S. Viral proteins were quantified using ImageJ software. (**B**) The histone H3 acetylation site of NbHAG1 was identified by LC-MS/MS, and the acetylation site is shown in red. (**C**–**E**) LC-MS/MS spectra of histone H3 acetylated peptides of CRISPER NbHAG1 and OENbHAG1 (*, *p* < 0.05). (**F**) Western blot detection of the protein content of H3K36ac in *NbHAG1* knockout mutants, WT and *NbHAG1* overexpression mutant plants. The protein content in the different samples was then determined by Ponceau S. Viral proteins were quantified using ImageJ software.

Modification of chromatin by HATs plays a key role in transcriptional regulation. Changes to histone acetylation, both at the gene promoter and in coding regions, correlates with transcriptional activation and repression [27]. Previous studies have shown that histone acetylation is both a cause and a consequence of transcription and that HAG1 can acetylate multiple histone H3 lysine residues [14,28]. Here, we detected the levels of H3ac in CRISPR NbHAG1, OENbHAG1 and WT plants, and found that the level of H3ac was significantly greater in OENbHAG1 plants than in the WT (Figure 4A). To test the direct modification site of NbHAG1, we performed Q-Exactive liquid chromatography-tandem mass spectrometry (LC-MS/MS) analysis. The results showed that in CRISPR NbHAG1 samples, the acetylation intensity of H3K36ac was significantly reduced compared to WT, whereas in OENbHAG1 samples, the acetylation intensity of H3K36ac was significantly elevated (Figure 4B–E, Tables S2 and S3). We subsequently validated this result with an antibody specific for H3K36ac, which demonstrated that NbHAG1 can indeed significantly affect the acetylation level of H3K36 (Figure 4F). These findings highlight the direct influence of NbHAG1 on H3K36ac levels, underscoring its role in chromatin modification and transcriptional regulation.

### 2.4. NbHAG1 May Regulate the Expression Levels of a Series of Genes

**Figure 5:**
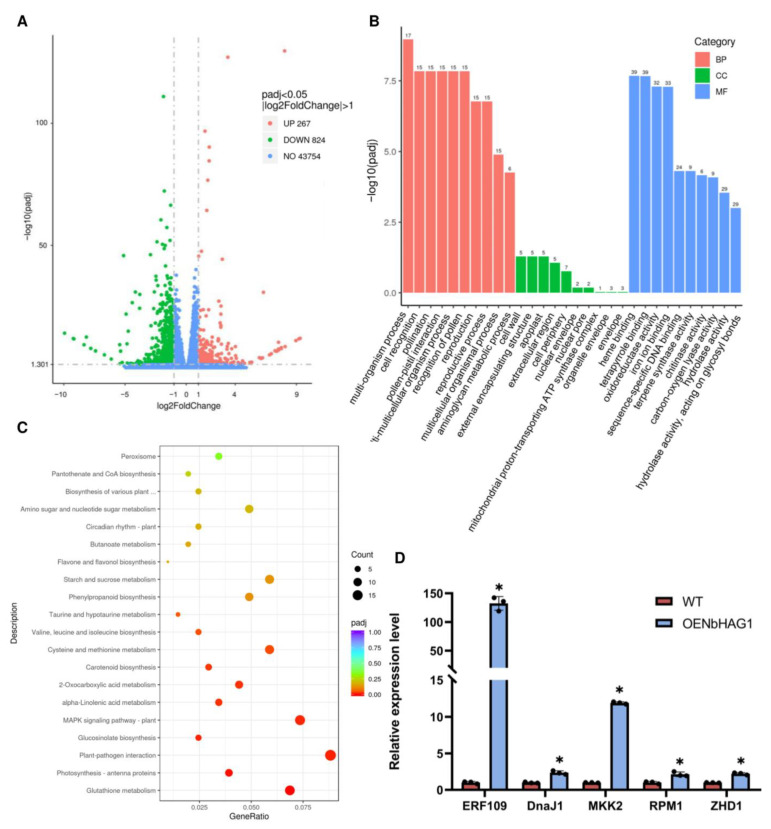
NbHAG1 may regulate the expression levels of a series of genes. (**A**) Volcano plots of DEGs between OEH and Nb. The two vertical dotted lines are twice the difference threshold, and the horizontal dotted line represents a *p*-value of 0.05. The red dots indicate the up-regulated genes in this group, the green dots indicate the down-regulated genes in this group, and the blue dots indicate the non-significantly differentially expressed genes. (**B**) Gene Ontology (GO) enrichment analysis of differentially expressed genes (DEGs) in OEH vs Nb, the top 10 significantly enriched terms in each primary are displayed. (**C**) KEGG pathway enrichment analysis of differentially expressed genes (DEGs) between the OEH and Nb. The left Y-axis represents the top 20 pathways. The X-axis represents the percentage of DEGs belonging to the corresponding pathway. The sizes of bubbles represent the number of DEGs in the corresponding pathway, and the colors of the bubbles represent the enrichment *p*-value of the corresponding pathway. (**D**) Validation of the DEGs by qRT-PCR. qRT-PCR was used to analyze the five genes in the disease resistance-related pathway that were significantly up-regulated in the overexpression mutants. Data presented are the mean ± SD of three biological samples per treatment. Each biological sample had three technical replicates. Significant differences between treatments were determined using Student’s *t* test (*, *p* < 0.05).

Since acetylation correlates with the transcriptional level of genes [28], we performed a transcriptome analysis on overexpression mutant (OEH) and control (Nb) plants. Those genes with corrected *p*-value < 0.05 and absolute fold change ≥2 were considered to be differentially expressed genes (DEGs). The differences in OEH vs. Nb were then evaluated using volcano plots. A total of 267 genes were up-regulated and 824 genes were down-regulated among all the DEGs found in the OEH vs. Nb comparison (Figure 5A, Table S4). The significantly enriched (*p*-value < 0.05) DEGs consisted of 274 GO terms in biological processes, 55 terms in cellular components and 183 terms in molecular functions. As shown in Figure 5B, the DEGs were significantly enriched in the multi-organism process, cell recognition and pollination during biological processes. Under cellular components, the DEGs were significantly enriched in the cell wall, external encapsulating structure, and apoplast. Under molecular function, the DEGs were significantly enriched in cell communication, heme binding, and tetrapyrrole binding (Figure 5B, Table S5). All DEGs were successfully assigned to 90 KEGG pathways and were significantly enriched in glutathione metabolism, photosynthesis-antenna proteins, and the plant–pathogen interaction pathway, as well as the MAPK signaling pathway (Figure 5C, Table S6).

Since overexpression of *NbHAG1* inhibited CWMV infestation, we hypothesized that NbHAG1 achieves disease resistance by regulating downstream genes, so we screened for disease resistance genes that were significantly up-regulated in the overexpression mutants. To further verify the reliability of the transcriptome data, we selected five genes that were most significantly up-regulated in the overexpression mutants and detected their expression in the mutants and controls by qRT-PCR. The results showed that the expression levels of the five genes were in good consistency with those detected by the transcriptome. Consequently, these results confirmed that the data we obtained from transcriptome analysis were trustworthy (Figure 5D, Table S7).

### 2.5. NbHAG1 Mediates H3K36ac to Regulate Downstream Gene Expression

**Figure 6:**
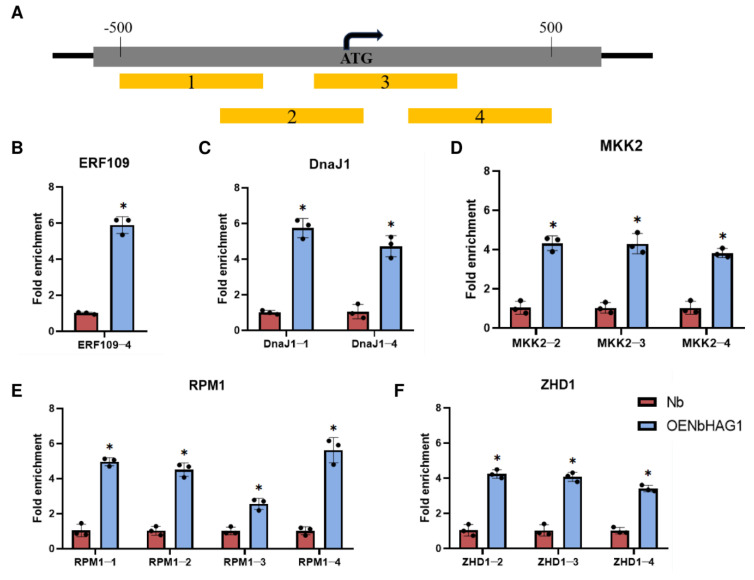
NbHAG1 mediates H3K36ac to regulate downstream gene expression. (**A**) Schematic representation of the fragment between 500 bp before and 500 bp after the downstream gene TSS. The fragments between 500 bp before and after the downstream gene TSS were divided into 4 small fragments, named 1–4 fragments. (**B**–**F**) Cut&Tag-qPCR to analyze the enrichment of H3K36 marks at the fragment between 500 bp before and 500 bp after the downstream gene TSS in *NbHAG1* overexpressing wheat plants. The enrichment levels were compared with DNA spike-in for enrichment detection. Data presented are the mean ± SD of three biological samples per treatment. Each biological sample had three technical replicates. Significant differences between treatments were determined using Student’s *t* test (*, *p* < 0.05).

Arabidopsis HAG1 has been shown to be an H3K36 acetyltransferase and has been strongly suggested to be the major enzyme depositing H3K36ac in vivo. Genome-wide chromatin immunoprecipitation sequencing experiments revealed that the peak of H3K36ac peaks at the 5′ end of genes, 199 nucleotides distal to the transcriptional start sites (TSS), independent of gene length [29]. Therefore, to explore how NbAHAG1 regulates gene expression, we designed four pairs of specific primer pairs in the fragment located between 500 bp before and 500 bp after the TSS (Table S8), and compared the H3K36ac levels of each fragment in OENbAHAG1 and wild type plants using Cut&Tag-qPCR (Figure 6A). In comparisons between OENbHAG1 plants and WT, there was a significant increase in H3K36ac enrichment for *NbERF109* in the region near the TSS (ERF109-4), for *NbDnaJ1* in the promoter region and in the region near the TSS (DnaJ1-1, DnaJ1-4), for *NbMKK2* in the promoter region and the region near the TSS (MKK2-2, MKK2-3, MKK2-4), for *NbRPM1* between the pre-TSS 500 bp and post-TSS 500 bp regions (RPM1-1, RPM1-2, RPM1-3, RPM1-4), and for *NbZHD1* in the promoter region and in the region near the TSS (ZHD1-2, ZHD1-3, ZHD1-4) (Figure 6B–F). These data confirm that NbHAG1 can indeed regulate downstream gene expression through acetylating the H3K36 site.

### 2.6. Silencing of NbERF109 and NbMKK2 Expression Inhibits CWMV Infection

**Figure 7:**
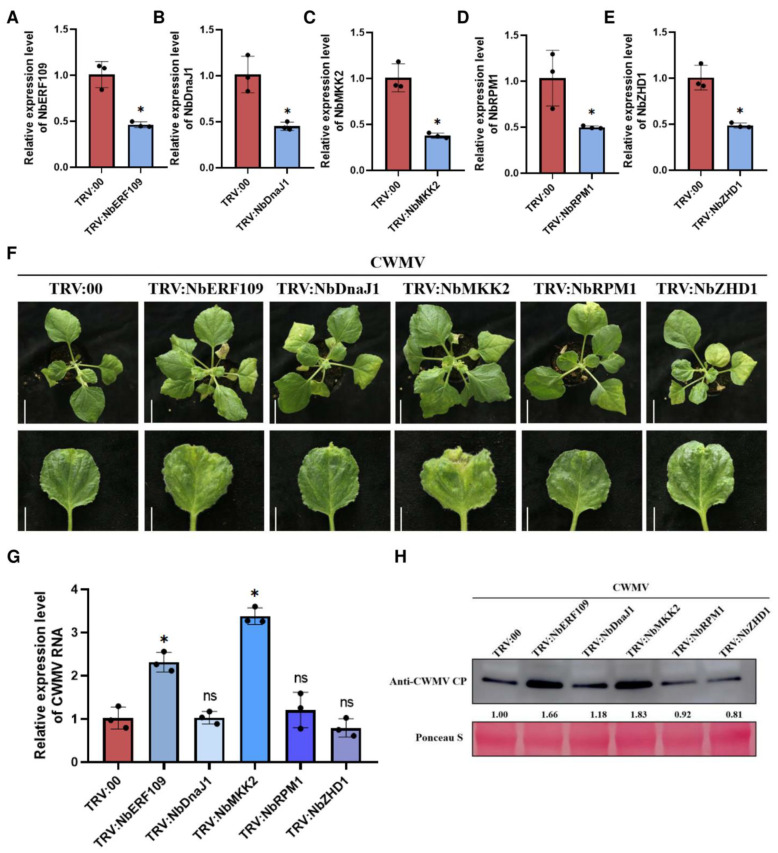
Silencing of *NbERF109* and *NbMKK2* expression inhibits CWMV infection. (**A**) Relative expression of *NbERF109* mRNA in the TRV:00- and TRV: NbERF109-inoculated *N. benthamiana* plants as determined through qRT-PCR. (**B**) Relative expression of *NbDnaJ1* mRNA in the TRV:00- and TRV: NbDnaJ1-inoculated *N. benthamiana* plants as determined through qRT-PCR. (**C**) Relative expression of *NbMKK2* mRNA in the TRV:00- and TRV: NbMKK2-inoculated *N. benthamiana* plants as determined through qRT-PCR. (**D**) Relative expression of *NbRPM1* mRNA in the TRV:00- and TRV: NbRPM1-inoculated *N. benthamiana* plants as determined through qRT-PCR. (**E**) Relative expression of *NbZHD1* mRNA in the TRV:00- and TRV: NbZHD1-inoculated *N. benthamiana* plants was determined through qRT-PCR. (**F**) Systemic mosaic symptoms on the CWMV-inoculated TRV:00, TRV: NbERF109, TRV: NbDnaJ1, TRV: NbMKK2, TRV: NbRPM1 and TRV: NbZHD1 plants. Photographs were taken 21 days post CWMV inoculation. Scale bars = 5 cm (upper panel) or 2 cm (lower panel). (**G**) QRT-PCR analysis of relative expression level of CWMV CP in the CWMV-inoculated TRV:00, TRV: NbERF109, TRV: NbDnaJ1, TRV: NbMKK2, TRV: NbRPM1 and TRV: NbZHD1 plants. (**H**) Western blot assay for CWMV CP accumulation in CWMV-inoculated TRV:00, TRV: NbERF109, TRV: NbDnaJ1, TRV: NbMKK2, TRV: NbRPM1 and TRV: NbZHD1 plant leaves. The protein content in the different samples was then determined by Ponceau S. Viral proteins were quantified using ImageJ software. Data presented are the mean ± SD of three biological samples per treatment. Each biological sample had three technical replicates. Significant differences between treatments were determined using Student’s *t* test (*, *p* < 0.05, ns, not significant).

Next, to investigate the role of downstream genes in CWMV infection, we first separately silenced the expression of *NbERF109*, *NbDnaJ1*, *NbMKK2*, *NbRPM1*, and *NbZHD1* in *N. benthamiana* plants using TRV-based VIGS technology. At 7 dpi, qRT-PCR results showed that *NbERF109*, *NbDnaJ1*, *NbMKK2*, *NbRPM1*, and *NbZHD1* were all successfully silenced in the corresponding infected plants (Figure 7A–E). These plants were then re-inoculated with CWMV. At 21 dpi with CWMV, the *NbERF109*-silenced plants (TRV: NbERF109) as well as the *NbMKK2*-silenced plants (TRV: NbMKK2) had more severe mosaic symptoms than the control plants (TRV: 00) (Figure 7F). A total of 14 days after infection with CWMV, qRT-PCR results showed that the levels of CWMV RNA accumulation were significantly higher in TRV: NbERF109+CWMV and TRV: NbMKK2+CWMV inoculated plants compared with TRV: 00+CWMV inoculated plants (Figure 7G). Western blot results were consistent with qRT-PCR results showing enhanced CWMV infection in *NbERF109*- and *NbMKK2*-silenced plants, but not in plants where *NbDnaJ1*, *NbRPM1* or *NbZHD1* were silenced (Figure 7H). However, how the downstream genes *NbERF109* and *NbMKK2* specifically inhibit CWMV infection and the molecular pathways by which they are involved in regulating the host defense mechanism against the virus need to be further investigated afterwards.

## 3. Discussion

HATs play essential roles in regulating chromatin structure, gene expression, plant growth, development, and stress responses [30,31]. HAT gene families have been identified and analyzed in several plants, including *Arabidopsis thaliana* [12], rice [32], barley [33], tomato [34], and wheat [25]. However, the specific functions of HATs in *N. benthamiana* remain largely unknown. We systematically identified nine *NbHAT* members in the *N. benthamiana* genome and categorized them into six clades similar to *AtHATs* and *TaHATs*, suggesting that the genes in each clade may have similar functions in all 3 plants (Figure 1). Previous studies have shown that from 7 dpi to 16 dpi, the expression levels of *TaHATs* continued to rise with increasing CWMV accumulation [25]. Our analysis showed that the expression of the acetylase *NbHAG1* was significantly higher than that of other acetylases after 21 days of CWMV infestation, suggesting that there may be a significant role for NbHAG1 in the CWMV infection process (Figure 2).

It has been shown that N-acetylation is a frequent protein modification essential for viability and stress responses in animals and plants [35,36]. We investigated the effect of the acetylase NbHAG1 on CWMV infection and showed that NbHAG1 plays a positive regulatory role in the defense response to CWMV (Figure 3). However, NbHAG1 did not interact directly with CWMV proteins (Figure S2), and we therefore speculate that NbHAG1 may affect downstream genes through acetylation to achieve resistance to CWMV infection. The results showed that in the case of changes in the expression of the acetylase *NbHAG1*, the expression levels of a series of downstream genes were also changed, including plant–pathogen interactions and plant MAPK signaling pathways related to disease resistance (Figure 5), and we therefore suggest that the resistance of NbHAG1 to CWMV is achieved through the regulation of downstream disease resistance genes. N-terminal acetylation of the βC1 protein encoded by the beta satellite of tomato yellow leaf curl China virus is critical for its viral pathogenicity and removal of N-acetylation of TYLCCNB-βC1 attenuated tomato yellow leaf curl China virus-induced symptoms [37]. As an acetylase, NbHAG1 must be able to affect the level of histone acetylation, so we believe that NbHAG1 regulates downstream resistance genes through mediating acetylation to activate the host’s defense response.

Induction of immune response genes during plant-pathogen interactions is usually associated with increased histone acetylation levels [38]. Here, we found that NbHAG1 as a histone acetyltransferase can activate the expression of a series of downstream disease resistance genes by elevating the level of H3K36ac (Figure 6). It has been shown that the histone acetyltransferase TaHAG1 interacts directly with a plant-specific zinc dependent protein TaPLATZ5 to potentiate the expression of TaPAD4 by increasing the levels of histone H3 acetylation and positively contributes to powdery mildew resistance via promoting SA and ROS accumulation in wheat [39].

We demonstrated that NbHAG1 can promote the expression of *NbERF109* and *NbMKK2* by mediating H3K36ac and found that silencing of NbERF109 and NbMKK2 followed by silencing of *NbERF109* and *NbMKK2* could promote CWMV infestation (Figure 6 and Figure 7). Ethylene-responsive transcription factor 109 (ERF109) is a member of the ERF/AP2 family of transcription factors, one of the largest families of transcription factors in the plant kingdom [40]. ERFs have been shown to play key roles in plant immunity. MAPK cascades are important for plant signal transduction, as they are involved in signaling of hormones, growth factors, microbes, or damage-associated molecular patterns, and they convert extracellular stimuli into intracellular responses while amplifying the transmitting signal [41]. Therefore, we believe that the resistance of NbHAG1 to CWMV is achieved by promoting the expression of *NbERF109* and *NbMKK2*, the mode of action of which needs further study. In addition, in our experimental results, silencing of *NbDnaJ1*, *NbRPM1*, and *NbZHD1* did not significantly promote or inhibit CWMV invasion. In addition, the NbHAG1 mutation did not have any effect on the growth and development of the plants. Therefore, we hypothesized that the expression of these three genes regulated by NbHAG1 was involved in other aspects such as the resistance against other viruses or the adaptation to the environment. NbHAG1 acts as an acetylase that regulates the acetylation level of histone H3K36, thereby sparing histone chromatin and exposing more gene-binding sites, allowing transcription factors to better recognize downstream genes.

**Figure 8:**
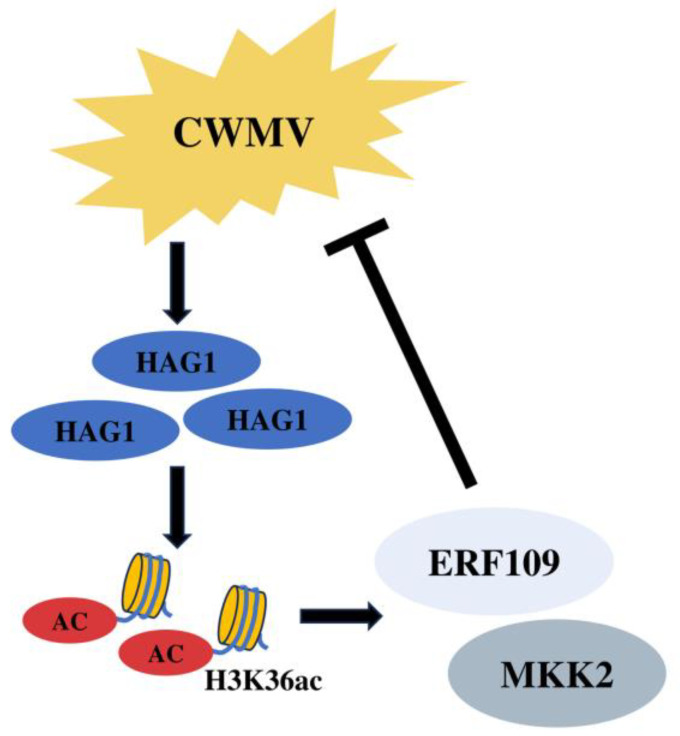
Working model showing the regulatory role of histone acetylase NbHAG1 in the activation of disease resistance genes by modulating histone H3 lysine 36 acetylation (H3K36ac). Following CWMV infection, there is a notable up-regulation in *NbHAG1* expression. As an acetylase, NbHAG1 significantly enhances the acetylation status of H3K36ac, thereby promoting the activation of downstream genes such as *NbERF109* and *NbMKK2*. This activation cascade effectively suppresses CWMV infection.

In summary, the histone acetyltransferase NbHAG1 can activate the expression of relevant genes in a series of downstream disease resistance pathways by mediating H3K36ac, among which NbERF109 and NbMKK2 can affect CWMV infection (Figure 8). Other targets of NbHAG1 and the specific molecular mechanism of post-transcriptional modification mediated by NbHAG1 during CWMV infection, as well as how it affects viral replication, transmission and host defense response, remain to be further studied.

## 4. Materials and Methods

### 4.1. Plant Materials, Growth, and Virus Inoculation

*N. benthamiana* seeds (from Shanghai Jinchao Technology Development Co., Ltd. Shanghai, China) were germinated in a growth chamber at 25 °C and 70% relative humidity under long-day conditions (16 h light/8 h dark cycles) [42]. Plants were inoculated as previously described and mock-inoculated plants were used as controls. After inoculation with CWMV, the plants were grown inside a climate room maintained at 15 °C until further analysis. *Agrobacterium tumefaciens* GV3101 harboring CWMV RNA1 (pCB-35S-R1) or RNA2 (pCB-35S-R2) infectious clones were obtained from a previously reported source (Yang et al., 2016). The mutant CWMV RNA2 constructs produced in this study were also transformed individually into *A. tumefaciens* GV3101, and the *Agrobacterium* cultures were grown individually overnight at 28 °C in a yeast extract peptone medium containing kanamycin (50 µg/mL) and rifampicin (50 µg/mL). The resulting Agrobacterium cultures were pelleted and then resuspended in an infiltration buffer (100 mM MES, pH 5.2, 10 mM MgCl2, 200 mM acetosyringone) to obtain an OD600 of 0.6 followed by >2 h incubation at 25 °C. *Agrobacterium* harboring pCB-35S-R1 was mixed with *Agrobacterium* harboring pCB-35S-R2 in a 1:1 ratio or with one of its derivatives prior to infiltration into N. benthamiana leaves. The infiltrated plants were grown inside a growth chamber maintained at 15 ± 2 °C, with a 14 h/10 h (light/dark) cycle and 70% relative humidity. The CWMV infectious clones were obtained as previously reported [23]. Samples were collected 14 days after inoculation and stored at −80 °C for further study. For all treatments and controls, there were three biological replicates per sample. Each experiment was repeated at least three times.

### 4.2. Genetic Transformation of N. benthamiana

Ten *N. benthamiana* seeds(from Shanghai Jinchao Technology Development Co., Ltd. Shanghai, China) were washed with 75% alcohol for 1 min, then sterilized using 15% H_2_O_2_ for 15 min, after which they were washed three times using water for 3 min each time, spread on MS medium (332 mg/L CaCl_2_ + 170 mg/L KH_2_PO_4_ + 1900 mg/L KNO_3_ + 180 mg/L MgSO_4_ + 0.83 mg/L KI + 8.6 mg/L ZnSO_4_)in an ultra-clean bench, and cultured in a light incubator (GXZ-1000, Ningbo Jiangnan Instrument Factory, Ningbo, China) at 28 °C for about 15 d. *N. benthamiana* leaves were cut into 0.5 × 0.5 cm^2^ slices on an ultra-clean table; the *N. benthamiana* leaves were transferred to the treated *Agrobacterium* spp. solution (OD value of about 0.5,CRISPR-NbHAG1 use CRISPR-Cas9-2gRNA-NbHAG1, OE-NbHAG1 use pCAMBIA35S-EGFP-NbHAG1 ), and infiltrated for 5 min; the leaves were blotted dry on a sterile filter paper, and then inoculated into MS solid co-culture medium (1/2MS + 30 g/L sucrose + 8 g/L agar, pH 5.8) and put in the dark at 22 °C in a constant temperature box for 2 d. The leaves on the plate were transferred to an induction medium (MS + 0.5 mg/L BA + 30 g/L sucrose + 8 g/L agar + 500 mg/L cef + 100 mg/L Kan, pH 5.8), so that the wound was attached to the surface of the medium with the adaxial surface facing upwards, and after transferring, the leaves were sealed with a sealing film and put into a light incubator to be cultured for 15 d. After a transfer to the same medium at about 15 d, the leaves on the plate were then transferred to a light medium (MS + 0.5 mg/L BA + 30 g/L sucrose + 8 g/L agar, pH 5.8) with the wound attached to the surface of the medium, face up. When small shoots grew on the induction medium, they were transferred to rooting medium (MS + 0.1 mg/L NAA + 30 g/L sucrose + 8 g/L agar + 100 mg/L Kan, pH 5.8) and cultured under light conditions for 40 d to grow shoots and induce rooting.

### 4.3. RNA Extraction and Quantitative Reverse Transcription-PCR (qRT-PCR) Assays

Total RNA was extracted from tissue samples using the HiPure Plant RNA Mini Kit (Magen, Guangzhou, China) following the manufacturer’s instructions and stored at −80 °C until use. A First Strand cDNA Synthesis Kit (Toyobo, Kita-ku, Osaka, Japan) with random primers was used to synthesize first-strand cDNA, and 1 µg total RNA was added per 20 µL reaction volume. Quantitative PCR was carried out using the SYBR Green qRT-PCR kit (Vazyme, Nanjing, China) on an ABI 7900HT sequence detection system (Applied Biosystems QuantStudio 5, Foster City, CA, USA) to detect the relative expression levels of the genes. At least three biological replicates and three technical replicates were used for all qPCR analyses. Each experiment was repeated at least three times. Relative expressions of the assayed genes were calculated using the 2^−ΔΔCt^ method. The primers used in this study are listed in Table S8.

### 4.4. Western Blot Assay

For total protein extraction, tissue samples were homogenized in a lysis buffer containing 60% SDS, 100 mM Tris-HCl (pH 8.8) and 2% β-Mercaptoethanol. Protein samples were analyzed in SDS-PAGE gels through electrophoresis (precast protein plus gel, 12%, 10 wells, HEPES Tris, Yeasen, Shanghai, China), and then transferred onto nitrocellulose membranes. The blots were incubated in a blocking buffer (5% skimmed milk and 0.05% Tween 20 in 1 × TBS) for 60 min followed by detection using specific primary antibodies and then an HRP-conjugated anti-mouse or anti-rabbit secondary antibody (TransGen Biotech, Beijing, China). Detection signals were visualized using an Amersham Imager 680 machine (GE Healthcare BioSciences, Pittsburgh, PA, USA).

### 4.5. Yeast Two-Hybrid Assay

Yeast two-hybrid assays were performed following the TaKaRa protocol handbook using strain yeast Y2H Gold. To investigate the interaction between NbHAG1 and CWMV proteins, the coding sequences of NbHAG1 and CWMV proteins were cloned to the Gal DNA-binding domain (vector: pGBKT7) or Gal4 activation domain (vector: pGADT7), respectively, and yeast two-hybrid assays were performed using primers listed in Table S8. Yeast cells carrying the co-transformed plasmids were plated onto a low-stringency selective medium lacking leucine and tryptophan (SD/–Leu–Trp) for 72 h to confirm the positive transformation and plated onto a high-stringency selective medium lacking leucine, tryptophan, histidine, and adenine (SD/–Leu–Trp–His–Ade) for 3 to 5 days to analyze the interaction. Yeast cells co-transformed with AD-T+BD-Lam or AD-T+BD-53 were used as controls. SD/–Leu–Trp(PM2220-20g), 1.19g/L, autoclave at 115 for 20 min, SD/–Leu–Trp–His–Ade(PM2110-20g), 1.15 g/L, autoclave at 115 for 20 min.

### 4.6. Bioinformatics Analysis of Differentially Expressed Genes (DEGs)

The Uniprot-GOA database (http://www.ebi.ac.uk/GOA/, accessed on 15 November 2023) was used for Gene Ontology (GO) annotation. Proteins were classified into three categories according to the GO annotation—biological processes, molecular functions, and cellular compartments. The Kyoto Encyclopedia of Genes and Genomes database KEGG (http://www.genome.jp/kegg/genes.html, accessed on 15 November 2023) was used for KEGG annotation [43]. The pathways of the identified DEGs were annotated using it and the annotation results were mapped on the KEGG pathway database using the Kyoto Encyclopedia online service tool KEGG Mapper. The enrichment results of GO terms and KEGG pathways were employed using a two-tailed Fisher’s exact test. The *p*-value was used to obtain significant enrichment GO terms and KEGG pathways, and *p* < 0.05 was considered to indicate statistical significance.

### 4.7. Virus-Induced Gene Silencing (VIGS)

To silence the expression of *NbERF109*, *NbMKK2*, *NbDnaJ1*, *NbRPM1* and *NbZHD1* in *N. benthamiana*, a 300 bp fragment representing part of their sequences was inserted into the TRV2 vector [44]. *Agrobacterium* culture carrying pTRV1 and pTRV2 (referred to as TRV:00), pTRV1 and pTRV2:NbERF109 (TRV:NbERF109), pTRV1 and pTRV2:NbMKK2 (TRV:NbMKK2), pTRV1 and pTRV2:NbDnaJ1 (TRV:NbDnaJ1), pTRV1 and pTRV2:NbRPM1 (TRV:NbRPM1), or pTRV1 and pTRV2:NbZHD1 (TRV:NbZHD1) was infiltrated into the leaves of *N. benthamiana* plants, and the infiltrated plants were grown inside a climate growth chamber at 25 °C for 7 days and then inoculated again with CWMV through agroinfiltration and maintained at 15 °C. Plants inoculated with TRV:00 were used as controls.

### 4.8. Identification of Acetylation Site in NbHAG1 Histone H3 through LC-MS/MS

Total proteins were extracted from plants overexpressing NbHAG1, separated by SDS-PAGE gel, and the expression of histone H3 was detected by electrophoresis and Coomassie Brilliant Blue staining with WT *N. benthamiana* plants as controls. The histone H3 protein band was cut from the gel and trypsin digested at 37 °C overnight, followed by LC-MS/MS. The H3 peptides were dissolved and separated using a reversed phase analytical column (Acclaim PepMap RSLC C18 column, Thermo Fisher Scientific, Waltham, MA, USA). The gradient phase was composed of 2 to 10% solvent (0.1% formic acid in 98% acetonitrile) in 6 min, 10 to 20% in 45 min, 20 to 80% in 7 min and holding at 80% for at least 4 min, all at a flow rate of 250 nL/min on an UPLC system. The peptides were subjected to ESI/NSI sources followed by MS/MS in Q ExactiveTM Plus (Thermo Fisher Scientific) coupled online to UPLC. The Orbitrap was used to detect whole peptides and ion fragments at a resolution of 70,000 and 17,500, respectively, with NCE set at 30. The electrospray voltage was set at 2.0 kV. Automatic gain control (AGC) was used to prevent overfilling of the ion trap. The m/z range was from 350 to 1800 for MS scans. The MS fixed first mass was set at 100 m/z and LC–MS/MS analyses were conducted at Micrometer Biotech Company (Hangzhou, China). The resulting raw data were processed using MaxQuant with integrated Andromeda search engine (v.1.5.2.8). Tandem mass spectra were searched against the same database. At the same time, the peptide was cleaved with trypsin. The number of peptide deletions was controlled to be less than 4, and each peptide had 5 modifications and 5 charges. The quality error of search was approximately 10 ppm, the main search error was approximately 5 ppm, and the error of fragment ions was approximately 0.02 Da.

### 4.9. Isolation of Nuclei and Cut&Tag

Nuclear and cytosolic proteins were fractionated following the protocol of the CelLytic PN Isolation/Extraction kit (Sigma-Aldrich, St. Louis, Missouri, USA). Briefly, total proteins were prepared from three replicate samples each of 2 g leaf tissues of the wild type (control, WT) and *NbHAG1* transgenic (OEH) plants with 400 μL protein extraction buffer and incubated on ice for 20 min followed by filtering through Miracloth (Calbiochem, Shanghai, China), and 40 μL solution was saved as total protein. The protein was centrifuged at 12,000× *g* for 10 min (5427R, Eppendorf, Hamburg, Germany), and supernatant was transferred into another 1.5 mL tube as soluble fraction. The sediment was washed by protein extraction buffer five times for 5 min each, and then resuspended in 40 μL nuclear protein extraction buffer. The collected nuclei were stained with 10 μg/mL 4′,6-diamidino-2-phenylindole (DAPI) and observed using a Leica TCS SP8 confocal laser scanning microscope (Leica Microsystems, Heidelberg, Germany). CUT&Tag assay was performed using NovoNGS CUT&Tag 4.0 High-Sensitivity Kit with DNA Spike-in control (N295-YH01, Novoprotein, Suzhou, China) as indicated. The detailed procedures were as previously described [45,46]. Briefly, protoplasts of 2-week-old NbHAG1 transgenic (OEH) plants and WT (CUT&Tag control) seedlings were isolated and incubated with NovoNGS ConA beads. The bead-bound protoplasts were incubated in primary antibody buffer containing an anti-H3K36ac antibody (39379, Active Motif, California, USA) at 4 °C by rotating overnight. After being washed three times, cells were treated with secondary antibody for 1 h. Subsequently, samples were incubated in pAG-Tn5 for 1 h. DNA tagmentation was performed at 37 °C for 1 h and the reaction was stopped with 10% SDS at 55 °C for 10 min. Following extraction with phenol-chloroform and ethanol precipitation, the DNA fragments were extracted, purified, and subjected to library construction using the reagents provided in the kit. Antibodies used in this study for CUT&Tag were H3K36ac (39379, Active Motif, California, USA), IgG or rabbit IgG (10500C, Invitrogen, Waltham, USA).

## 5. Conclusions

In this study, we elucidate the role of acetylase NbHAG1 in the mechanism underlying infection by the CWMV, contributing to a deeper comprehension of acetylase involvement in viral host infection. Our experimental findings demonstrate that NbHAG1 serves as a crucial facilitator of resistance against CWMV infection, orchestrating the activation of transcription for downstream disease resistance genes by augmenting the acetylation level of H3K36ac. This elucidation offers a novel theoretical framework for the management of CWMV infections.

## Data Availability

Data are contained within the article and Supplementary Materials.

## Supplementary Materials

The following supporting information can be downloaded at: https://www.mdpi.com/article/10.3390/ijms25052800/s1.
