# Supplementary material for: Regulation of Disease-Resistance Genes against CWMV Infection by NbHAG1-Mediated H3K36ac
Source: Int J Mol Sci. 2024 Feb 28;25(5):2800. doi: 10.3390/ijms25052800 (PMC10931728; doi:10.3390/ijms25052800)
Supplement: Supplementary file 1 [file ijms-25-02800-s001.zip › ijms-2871725-supplementary/Figure S1. Growth phenotypes of the wild-type and NbHAG1 mutant plants.pdf]

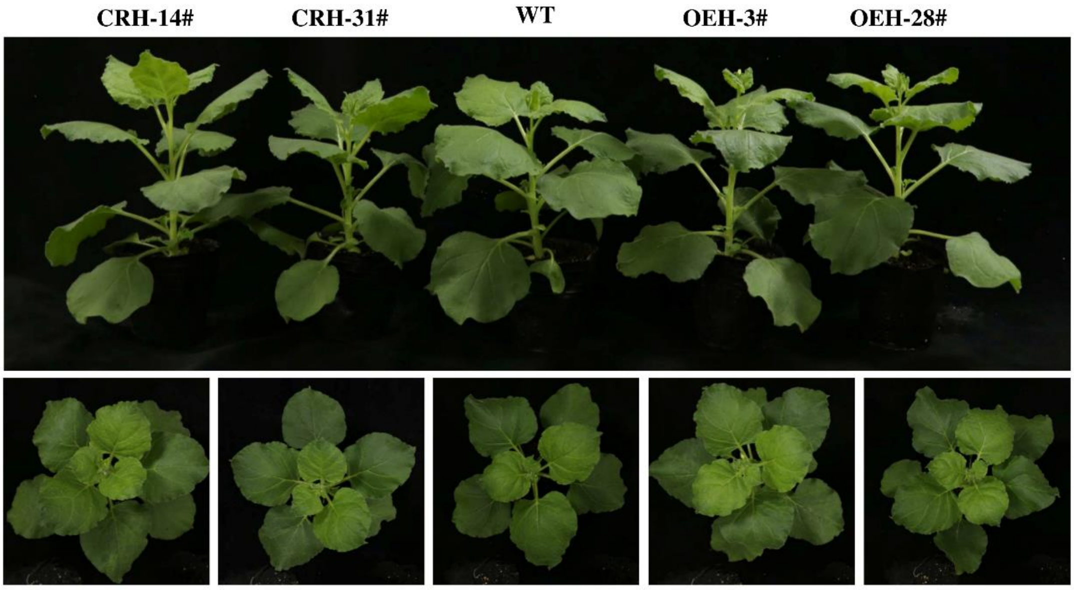

Figure S1. Growth phenotypes of the wild-type and NbHAG1 mutant plants after 30 days of growth in a growth chamber.
