# Supplementary material for: Regulation of Disease-Resistance Genes against CWMV Infection by NbHAG1-Mediated H3K36ac
Source: Int J Mol Sci. 2024 Feb 28;25(5):2800. doi: 10.3390/ijms25052800 (PMC10931728; doi:10.3390/ijms25052800)
Supplement: Supplementary file 1 [file ijms-25-02800-s001.zip › ijms-2871725-supplementary/Figure S2. No direct interaction between NbHAG1 and CWMV proteins.pdf]

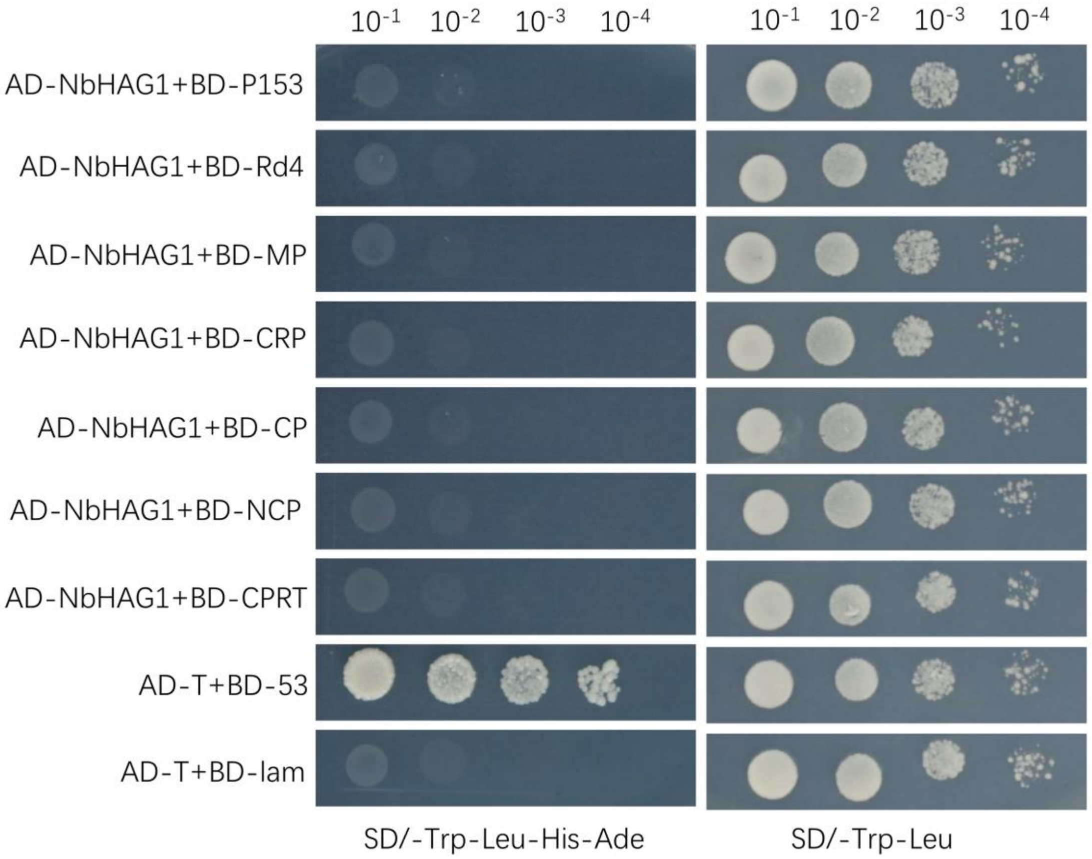

Figure S2. Yeast two hybrid assay showing that there is no direct interaction between NbHAG1 and the CWMV proteins：P153, Rd4, MP, CRP, CP, NCP and CP-RT. The transformed yeast cells were grown on SD/-Leu/-Trp medium and then on SD/-Trp/-Leu/-His/-Ade medium. Yeast cells co-expressing AD-T+BD-Lam or AD-T+BD-53 were used as controls.
